# Barriers to sharing qualitative interview data and suggestions to overcome them: a scoping review

**DOI:** 10.1098/rsos.261007

**Published:** 2026-10-28

**Authors:** Evangeline Gowie, Anna Tsakalaki, Halena Lad, Ruth Davies, Chloe Beesley, Etienne Roesch

**Affiliations:** ^1^ School of Psychology and Clinical Language Sciences, University of Reading, Berkshire, UK; ^2^ Centre for Integrative Neuroscience and Neurodynamics, University of Reading, Berkshire, UK; ^3^ School of Humanities, Social and Education Sciences, Department of Education Sciences, European University Cyprus, Nicosia, Cyprus; ^4^ Research Management and Innovation Directorate, King's College London, London, UK

**Keywords:** qualitative research, open data, research data management, qualitative data, consent, confidentiality, interview transcripts

## Abstract

Universities, journals and funding bodies increasingly encourage qualitative data sharing, yet practical guidance for making interview and focus group transcripts accessible remains difficult to locate. This scoping review maps actionable strategies to overcome barriers to sharing qualitative data. The review included all sources of evidence, enabling capture of suggestions made by qualitative scholars themselves, as well as those from journals and repositories. A comprehensive search of peer-reviewed and grey literature identified 110 sources. Extracted suggestions were categorized into seven interrelated themes: confidentiality, consent, data misuse, context, external approvals, researcher distress, and time and financial constraints. These findings are consolidated into a table-based tool to guide researchers toward the most practical resources for each barrier. The review highlights the diversity and complexity of qualitative data sharing, the absence of systematic evaluation of strategies and the importance of avoiding one-size-fits-all data sharing expectations. By providing a structured, accessible resource, this work aims to support researchers in ethically, effectively and confidently sharing qualitative data, while informing funders and institutions developing open data policies.

## 1. Introduction

Open research is a transdisciplinary movement focused on improving the accessibility of research methodology and outputs. As part of this effort, universities, academic journals and funding bodies are progressively encouraging the sharing of research data [1]. The rationale typically offered often focuses on replicability, emphasizing that accessible data allows others to scrutinize and validate conclusions through reanalysis. This rationale can alienate some qualitative scholars, as replicability is not always relevant to the constructivist paradigm from which their research is conducted (e.g. [1,2]). Nonetheless, many qualitative researchers support data sharing on alternative grounds. In particular, sharing qualitative data could address priorities of both participants and researchers to maximize the impact of individual contributions and reduce over-researching of specific populations [1,3–5].

As this philosophical debate around data sharing increasingly gains interest among qualitative researcher circles [4–8], so does a parallel discussion around practical implementation. Qualitative data are more complex to prepare for sharing than standard quantitative data, and generalized guidance does not account for this complexity. For example, a review published in 2024 found that of 257 social psychology journals that accept qualitative research, only three had explicit guidance on open qualitative data [1]. Qualitative researchers who feel that their data would be suitable for sharing, therefore, call for clearer, actionable guidance on how to do so ethically and usefully [1,5,9–11]. Such guidance does exist, but is dispersed across a range of sources, and often obscured by extensive philosophical discussion on the appropriateness of qualitative data sharing. Identifying actionable recommendations therefore takes time; a limited resource in the current academic climate where workloads are already at full capacity [12].

Thus, although qualitative researchers, participants, journals and funders often want to promote data sharing, clear guidance on how to do so ethically and effectively remains difficult to locate. The aim of our work is, therefore, to address this gap through a comprehensive scoping review that maps existing guidance across the literature. To do this, we extracted all actionable suggestions to overcome any identified barrier to qualitative data sharing and sorted them thematically by barrier. The review was limited to researcher-level actions, as the goal was to create a resource to support researchers. In light of this goal, we used the results to inform a table-based tool [13] to guide researchers through the literature to the most practically applicable resources for the specific barrier they are facing, thus reducing the overall time burden of sharing qualitative data. To that end, the research questions of this review are:

— What barriers to sharing qualitative data does the literature seek to address?
— What solutions have been suggested to overcome these barriers?

## 2. Materials and methods

This review was conducted according to the JBI guidance for scoping reviews [14], and is reported in accordance with the PRISMA extension for Scoping Reviews (PRISMA-ScR) checklist for transparent reporting of results [15].

We pre-registered the review on OSF: https://osf.io/3rjga/overview, but significant alterations were made during the research process. Most notably, after screening the literature, we opted to conduct a scoping review instead of the originally planned systematic review. Systematic reviews aim to formally evaluate the evidence base against a normative framework, focusing on minimizing the risk of bias [16]. Scoping reviews, in comparison, are appropriate when literature is complex and varied, and seek to map rather than evaluate key concepts and findings [14–16]. Our literature search revealed limited experimental evidence, and a high prevalence of reflective accounts of researchers attempting to share their own data. A systematic review seeking to appraise the evidence and minimize bias was, thus, deemed neither possible nor desirable. We, therefore, opted to conduct a scoping review, synthesizing concepts descriptively and omitting any formal evaluation.

### 2.1. Inclusion/exclusion criteria

Our pre-registered inclusion criteria were: any output that (i) identifies a barrier to sharing qualitative data, and (ii) suggests an action that researchers can take to overcome this barrier. However, during screening we adjusted this to specify that we are specifically interested in interview or focus group transcripts. This decision was made because the first sift revealed extensive literature on clinical notes, which was deemed outside the scope of this review. The final inclusion criteria were therefore: any output that (i) identifies a barrier to sharing interview or focus group transcripts, and (ii) suggests an action that researchers can take to overcome this barrier. No restrictions were placed on the type of source, date of publication, geographical location or discipline of the data to be included in this review.

The exclusion criteria were (i) no specific suggestions/solutions included in the paper (see Discussion), (ii) raw dataset; because it was not deemed within the scope to analyse no English language version available, because the extraction team only contained monolingual English speakers.

### 2.2. Search strategy

Peer-reviewed journal articles were located through the following online databases: PubMed, Web of Science, PsycINFO, and the Cochrane database of systematic reviews. Grey literature was located through OpenAIRE and the Bielefeld Academic Search Engine (BASE). Published articles were excluded while searching BASE. The following search string was manually adapted for each database: (‘Qualitative’ or ‘Field Notes’ or ‘interview’) and (‘open’ or ‘shar*’ or ‘repositor*’ or ‘archiv*’ or ‘reus*’). This is an updated version of the pre-registered search as screening revealed that our original terms were missing some keywords. We manually searched the reference lists of all included items for any additional eligible articles that were not captured in the initial database search. The search was conducted between 13 February 2023 and 4 June 2023. A repeat search was performed on 28 July 2025.

EG also contacted members of the UK Reproducibility Network special interest group on open qualitative research, who directed EG to useful sources. All sources provided were already included in the review.

### 2.3. Source of evidence selection

Details of the item selection process are outlined in figure 1. Items were initially imported into Rayyan [17,18]. Rayyan's ‘auto-detect duplicates’ feature was used to identify duplicates, and EG manually verified each identified pair. The title and abstract screening were conducted in Rayyan. First, 15% of articles were double-screened by EG and EW. There was a 98.4% agreement between screeners, leaving four conflicts, all relating to ‘open innovation’. Following discussions on conflicts between EG and EW, a consensus was reached that ‘open innovation’ did not fall within the scope of the review and all four were excluded. EG screened the rest of the titles and abstracts without a second screener. Articles that passed screening were imported into NVivo for full-text review.

Full texts were screened by two researchers. EG screened all sources, and a second screen was conducted by either CB, RD or HL. This was done alongside data extraction, with sources being marked as excluded if no specific action to overcome a barrier was identified.

### 2.4. Data extraction

Suggestions from each paper were double extracted by two authors (EG and either CB, RD or HL) using the qualitative data analysis software NVivo. *Suggestions* were defined as ‘any action that an individual researcher can take to overcome an identified barrier to sharing their transcripts'. Extractors read each included source in full and applied line-by-line coding to extract all suggested actions. Suggestions were extracted from all parts of source materials. To note, authors did not need to endorse the action for it to be extracted. When extractors identified a new suggestion, a new code was created for this suggestion. Subsequent references to the same suggestion were sorted into the existing code. Barriers were not collected unless accompanied by a corresponding suggestion to overcome them (see Discussion). Regular meetings were held to discuss and reach consensus about what counted as a suggestion.

### 2.5. Data analysis/presentation

Suggestions to overcome identified barriers to sharing qualitative data were analysed inductively, using descriptive content analysis [13]. EG, RD, CB and HL first read through sources independently to generate initial codes. These were suggested strategies to overcome a specific barrier, organized into second-order codes representing the barrier they sought to overcome. Regular calibration meetings were held between coders to discuss conflicts. Conflicts primarily revolved around what counted as an action that a researcher could take. For example, ‘take training’ was proposed as a code by two coders, and ultimately decided that it did not count as an actionable suggestion unless specific, accessible training was signposted. Following conflict resolution, coders reviewed their previous codes to align with consensus. EG then charted barrier codes into higher-order themes of barriers. Themes were discussed and refined with AT and ER. This refinement was limited to grouping second-order codes into themes, and so did not require recoding at any point. This paper provides a narrative summary of each theme, and practical information on individual suggestions is presented in a table-based tool for navigating the included literature: https://osf.io/d839q/files/v2ras. At no point was any sort of AI used in the extraction or analysis of the data.

## 3. Results

### 3.1. Description of included studies

In total, 5436 items were identified through the initial search. 110 met the inclusion criteria. Source types are described in table 1. All items were published between 1995 and 2025.

Suggestions were sorted into seven themes of barriers: confidentiality, consent, data misuse, context, external permissions, researcher distress, and time and money. Research participant-centred themes are discussed first, followed by researcher-centred themes and finally practical themes. We condensed these results into a table-based tool to direct researchers to the most informative reading for each suggestion. This tool is designed for ease of reading and therefore only contains the most helpful sources for each proposed strategy. A complete map of the references to each suggestion is included in the supplementary material, and this paper presents a narrative summary of each theme. Narratives provide an overview of the suggestions made and the level of guidance found around the suggestions. Reported counts represent the number of sources that reference each suggestion.

**Table 1.** Description of source types.

source type	*n*
opinion pieces (characterized as an item offering the author's opinion without reference to any specific experience of sharing qualitative data)	46
reflexive accounts of engaging with open data	30, 17 through working for an archive, 12 through sharing one's own data and one through using someone else's data
guidance articles	9
presentation outputs (slides or discussion summaries)	8
interview studies	6
articles assessing AI tools for assisting de-identification	6
literature reviews	3
blog post	1
example of shared data used as an example of a well-formatted contextual summary	1

**Figure 1.**
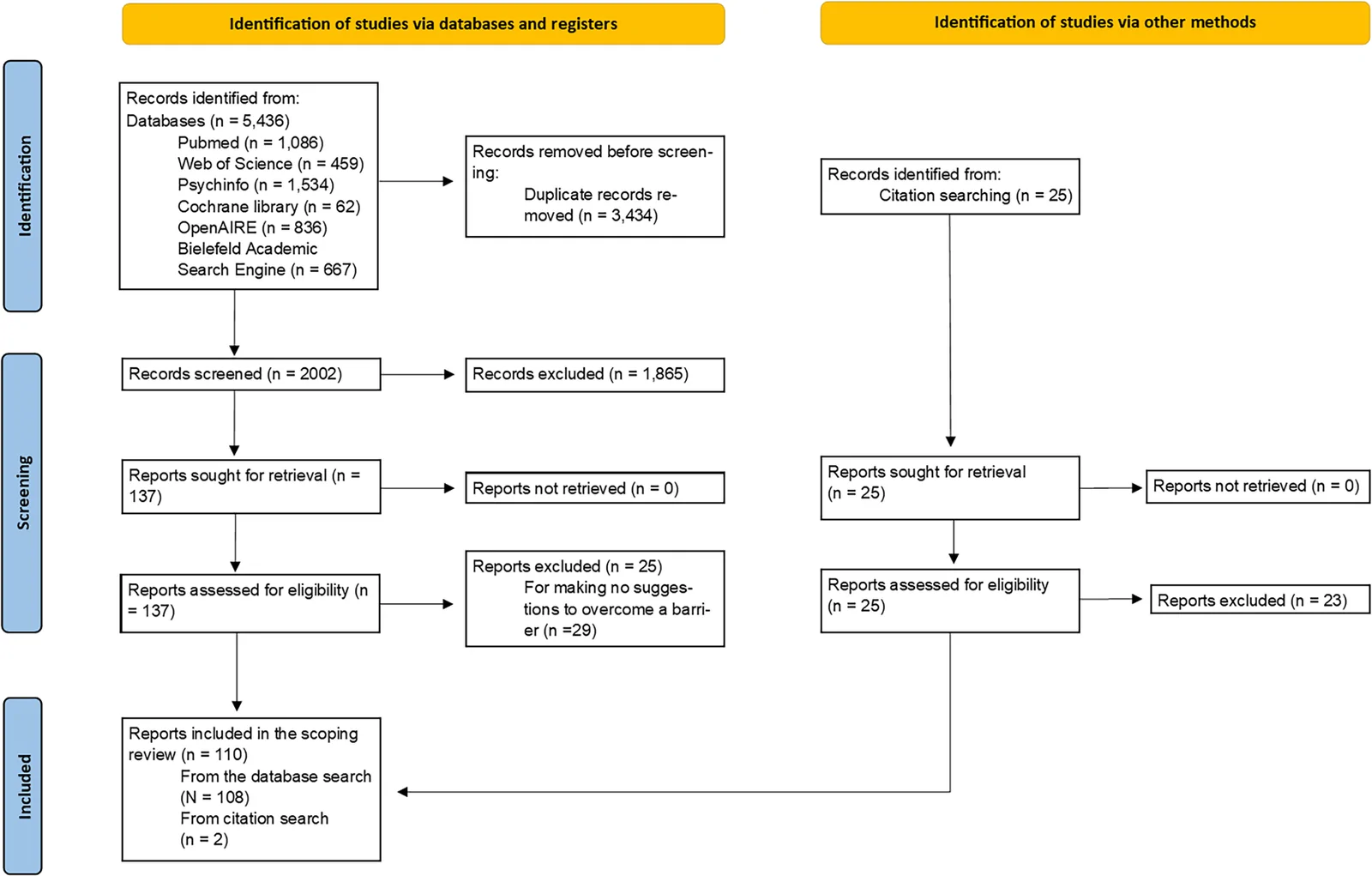
PRISMA diagram charting the selection process. ‘Records’ refers to source metadata (title, abstract, authors, etc.). ‘Reports’ refers to the full source.

#### 3.1.1. Theme 1: confidentiality

From this point onward, ‘authors’ refers to the authors of the sources we examined. Confidentiality is consistently referenced as a central challenge, with authors highlighting uncertainty surrounding both identifying potential identifiers and deciding how to obscure them without undermining the value of the data. All authors agreed that direct identifiers (names, addresses, etc.) should be removed unless participants explicitly request credit for their contribution. They also agreed that this would be reasonably simple, if time-consuming, to achieve. However, the issue becomes more complex when considering that transcripts may be read by ‘insiders’, who have personal knowledge of participants' stories. This means that ‘a minor detail—such as a manner of speaking or a specific phrase—that is of unknown significance to the investigator (and therefore likely to go unredacted) could result in deductive disclosure’ [19].

To facilitate effective de-identification, authors propose a structured de-identification process (*n* = 8) through which transcripts are divided into ‘data points’ [20], which are systematically examined for any items that should be considered for remediation. Suggested remediation tactics are removal (*n* = 12), blurring (by replacing specifics with categories, *n* = 16) or fictionalization (*n* = 12). This must be a highly involved and individualized process, akin to ‘a type of qualitative analysis in its own right’ [20]. Four sets of authors [21–24] discuss the process at a highly practical level, with details such as how and with whom the analysis protocol was developed [22], specific questions to ask oneself about each data point and the pros and cons of different analysis software. Three sets of authors [20,22,25–27] recommend that insider knowledge should be leveraged to reduce the risk that fellow insiders may identify participants. This could be achieved either by asking participants themselves or appropriate proxies.

Three sets of authors further suggest validating de-identification through ‘intruder testing’, whereby an allocated individual (either the researcher, a relevant expert or an independent third party) takes on the role of an intruder and attempts to identify participants from de-identified transcripts [20,21,28].

In addition, limiting the level of openness is commonly proposed as an additional protective measure should researchers remain uncomfortable that their transcripts are sufficiently de-identified. This could be through data embargo (*n* = 20), or placing limitations on the people or for what purpose one can see the data (*n* = 42). Different repositories offer different levels of access controls, and authors recommend confirming requirements in advance. Certain transcripts may remain too identifiable to be shared and should be removed in their entirety. Finally, two sources suggest minimizing identifiable information at the point of data collection [29,30].

#### 3.1.2. Theme 2: consent

Authors broadly agree that the traditional model of obtaining one-off consent for data sharing immediately before data collection does not sufficiently account for the inherent complexity of qualitative data. They emphasize that the exact steps that will be taken to protect a participant's identity are difficult to communicate in advance, and researchers are not always convinced that participants fully understand what they have consented to. For example, VandeVusse *et al.* [31] found that despite their best efforts to explain exactly what ‘data sharing’ means, 16 out of 64 participants ‘demonstrated an incomplete or inaccurate understanding’. In response, the researchers chose not to archive transcripts from these participants. They suggest that this approach could become a planned safeguard, whereby participants are asked to explain data sharing in their own words, and transcripts are excluded if a sufficient level of understanding is not demonstrated.

To address issues of understanding more generally, authors propose preparing a verbal script alongside the written consent form. A range of information that should feature in such scripts is discussed (*n* = 31), and 16 sources provide example language for either written consent forms or verbal discussion. Authors also recommend conducting common-sense risk assessments (*n* = 2), explaining that if participant behaviour suggests discomfort with data sharing, or if there is reason to believe that potential harms have not been considered, details should be removed regardless of consent.

Further, researchers are concerned that asking for consent for data sharing may negatively impact data quality, either by encouraging self-censorship or by discouraging participation altogether. Notably, the experiences of included authors suggest that requesting consent for data sharing is unlikely to prevent participation [4,8,27,32], although there have been no attempts to assess the impact on self-censorship. Proposed strategies to mitigate this concern centre around ensuring that participants feel they have control over their data. Commonly suggested measures include allowing participants to specify safeguards for their data (*n* = 3), providing transcripts for review with the option to redact details or sections (*n* = 3) and allowing participants to opt out of data sharing while remaining included in analysis (*n* = 6). One article suggests that not providing this option would be unethical as it would raise the barrier for participation disproportionately for vulnerable or marginalized populations, thereby pushing their stories out of the research process [2]. Additional suggestions to limit impacts on data quality are to ask for permission to share data only after the interview has completed (*n* = 4), and to provide a full explanation of the benefits of data sharing (*n* = 2).

To address consent-related concerns in general, authors propose a ‘consent as a process’ approach (*n* = 14), through which consent is re-addressed at multiple points during the research and finding dissemination process. Individual suggestions examine specifically how and when to discuss consent with participants to address each concern raised. Most agree that as a minimum, consent should be re-obtained at the end of an interview, but suggestions range from disclosing intent to ask for consent in recruitment information [26] through to asking for consent to share with each new audience identified (*n* = 2).

#### 3.1.3. Theme 3: data misuse

Authors highlight that data could be misappropriated in a way that harms a community or group even if individual participants are not identifiable. For example, Branney *et al.* [33] discuss concerns surrounding ‘quotes being taken out of context in the popular media to assign participants a certain membership category that might pathologize them, such as ‘illegal immigrants’. Importantly, concerns about data misuse do not solely pertain to deliberate or malicious actions. Hardy *et al.* [34] highlight concerns that secondary researchers may cause distress by reanalysing interviews focusing on an aspect of stories that participants had not consented to being examined. For instance, their participants consented to analysis focusing on resilience, but not for analysis focusing on addiction. Alternatively, secondary researchers may simply misinterpret narratives owing to the absence of contextual cues, such as facial expressions or other social signals that participants use to convey meaning [8], thus misrepresenting participants' experiences.

The most common suggestion to address concerns around data misuse is access controls (*n* = 15). This could be addressed on a case-by-case basis (*n* = 5), or through pre-specified conditions such as institutional credentials (*n* = 5), ethical approval (*n* = 2) or specifications on exactly what purposes data can be used for (*n* = 6). Consulting the original authors during the secondary analysis was also suggested to alleviate concerns about data misuse (*n* = 3), particularly through misinterpretation. Hardy *et al.* [34] felt that their safeguards were strengthened by consulting community insiders during the development of their data management plan. Finally, three sets of authors suggest that recontacting participants for consent at each new request for sharing would be best practice, although none claim that this is practically viable.

#### 3.1.4. Theme 4: context

Many authors discuss that simply sharing transcripts is insufficient to enable reuse, as additional contextual details are required to interpret meaning. There is broad agreement that the specific contextual information needed will vary by project. Forty-five sources make suggestions about individual details or materials that would assist a secondary researcher in understanding context. Common recommendations are reflexivity/field notes (*n* = 32), links to associated publications and other outputs (*n* = 26), interview schedules/topic guides (*n* = 14), a description of the interview setting (*n* = 13) and information about individual participants (*n* = 8). Many additional materials are also suggested by smaller numbers of sources. In addition, five frameworks outlining contextual information that should accompany any qualitative dataset are discussed and evaluated (*n* = 8).

Further, authors highlight a tension between protecting participant anonymity and providing sufficient contextual detail to enable reanalysis. Further, several note that removing unique identifiers may inadvertently obscure experiences specific to minority groups, adding an additional ethical layer over the practical considerations [23,35]. One proposed strategy for mitigating the impact of anonymization on secondary interpretations is to explicitly note the impact that detail remediation has on contextual information (*n* = 3). Sources offering this recommendation do not go into detail about how it should be done, suggesting only that a researcher may describe in broad terms what has been redacted and why [29], potentially via an anonymization log [11].

The second suggestion is to consider whether full anonymization is required in all cases. At the more extreme end of this suggestion, Walsh *et al.* [36] describe a participant who was particularly difficult to de-identify and who consented to the full sharing of their transcript. More commonly, authors suggest that it may not be necessary to remove all potential identifiers, as many would only become identifiers when combined with other contextual details (*n* = 5). Selecting potential identifiers to retain is recognized to be a complex and sensitive process. Two papers discuss the process in depth [22,23], one of which includes supplementary material detailing instructions given to de-identifiers for a large qualitative archiving project [22]. Additionally, the more stringent the access controls placed on a dataset, generally the less data it is deemed necessary to remediate (*n* = 6).

Finally, some authors contest that context can never be fully conveyed through written information alone. Collaboration between primary and secondary researchers may therefore be beneficial (*n* = 10). Four levels of collaboration are suggested: simply attaching contact information to the dataset (*n* = 1), requiring an onboarding process as a condition of data access (*n* = 2), interpretation checking by the primary researcher (*n* = 1), or including the primary researcher in the secondary analysis team (*n* = 1).

#### 3.1.5. Theme 5: obtaining external approval

The fifth barrier theme covers difficulties obtaining external approvals for qualitative data sharing. Authors discuss challenges associated with two forms of external approval: copyright approvals and ethics committee approval. Authors discuss uncertainty around the ownership of the copyright of qualitative data, explaining that as data are co-created and contain the participant's own words, participants may own the copyright of the data [37]. This could mean that researchers do not have the legal right to make decisions about whether to share data. The most commonly proposed solution to address this is to explicitly request that participants assign copyright to the researcher through the consent process (*n* = 7, based in the UK, the USA and Austria), although two sources (UK and USA) suggest that this may not be necessary under the ‘fair use’ exception whereby ‘in the absence of a written agreement, the researcher may use the interview data for purposes which have been explained to the respondent or for uses which might be ″reasonably″ expected’ [38]. We acknowledge that legal requirements vary between jurisdictions. Assessing these legal complexities was beyond the scope of this review, which instead reports the suggestions described in the literature.

Authors also discuss difficulty obtaining ethical approval when institutional review boards are not familiar with qualitative data sharing, and err towards risk-averse decisions [37,39–41]. Advice to obtain ethical approval is only provided by one paper, which outlines a four-step approach to obtaining institutional review board approval for qualitative data sharing [42]. The four steps are: (i) present a clear plan to document and systematically abide by participant views, (ii) emphasize confidentiality assurances, (iii) emphasize that sharing data where a participant requests it treats them as an autonomous agent, and (iv) highlight post-interview consent forms.

#### 3.1.6. Theme 6: researcher distress

The sixth theme concerns the researcher's internal distress as a barrier to data sharing. Three sources of distress were identified: a sense of moral responsibility to protect participants, vicarious trauma, and uncertainty about where to seek support.

Discussions of moral responsibility arise from reflective accounts of sharing qualitative data. Authors share two strategies that helped prevent these concerns from inhibiting data sharing. The first is to acknowledge and respect these feelings by abiding by common-sense risk assessments (*n* = 2), removing details or embargoing transcripts where they felt that to do otherwise would violate their own moral judgement. The second strategy is to engage participants in discussion about data sharing, thereby assuring themselves that they are adhering to participants' wishes [43].

Vicarious trauma refers to the psychological distress evoked by the de-identification process, which requires researchers to repeatedly reread distressing material from participants whom they may have developed a connection with. Suggested mitigation strategies relate exclusively to researchers working in a team to de-identify transcripts, emphasizing mutual support among team members. These were to enforce shifts to ensure breaks [21] and to provide check-ins [20] or signpost to emotional support resources (*n* = 2).

To address the issue that researchers do not know where to obtain support with qualitative data sharing, authors suggest a range of sources of support. The most common by far is repository staff (*n* = 15), particularly those from specialist qualitative data repositories.

#### 3.1.7. Theme 7: time and money

The final theme covers the constraint of time and financial resources required for anonymization. Collectively, the barriers associated with qualitative data sharing mean that preparation requires an amount of time and money that is perceived as prohibitive by many researchers.

Several strategies to reduce the resources required are suggested. Firstly, pre-planning de-identification (*n* = 7) can help avoid discovering institutional or repository requirements and restrictions after work has been conducted and therefore avoid having to repeat processes. Planning ahead also allows researchers the option of de-identifying transcripts as they create files for analysis, potentially reducing time required for repeated reading. Automated de-identification is also discussed (*n* = 19). Ten specific tools are identified as potentially suitable, and two papers provide comparative overviews that weigh the advantages and limitations of different tools. A final suggested strategy is to reduce the remediation required by increasing access restrictions (*n* = 2), sharing only a subset of transcripts (*n* = 1) or coding queries (*n* = 1), or seeking participant consent to share identifiable data (*n* = 2).

However, the most widely recommended approach to prevent time and financial requirements inhibiting data sharing is to incorporate these costs into grant applications (*n* = 20). Although no article offers specific guidance on the level of resources that should be requested, several reflective accounts did report the resources they used in the process:

— Campbell *et al.* [20]. ‘*The primary work was completed in 4 months, and we needed a fifth staff member to assist as our close-out date loomed*’ (32 interviews).
— Weller [44]. ‘*The resultant 145 transcripts and audio files of in-depth interviews, 500 activity sheets and contextual notes, required over 600 hours of preparation.*’
— Saunders *et al.* [23]. ‘*More than a month's labour was devoted to anonymising just 20 of the interviews (three weeks' full-time from the first author with additional input from the two co-authors*).’

## 4. Discussion

### 4.1. Summary

This scoping review sought to catalogue suggestions to overcome barriers to sharing interview transcripts. Of the 110 items included, most were opinion pieces or reflective accounts published in peer-reviewed journals.

Our pre-registered plan was to conduct a full systematic review, complete with quality assessment. However, the literature was more diverse than anticipated, with a very small proportion of sources being appropriate for quality assessment. A systematic review was therefore inappropriate. Instead, a scoping review was undertaken to map the current suggestions and direct readers to informative sources.

Extracted suggestions covered seven themes of barriers: confidentiality, consent, misappropriation of data, context, external approvals, researcher distress, and time and money. These themes align with those identified through surveys [4,45–47] and discussion groups [8]. We present our findings in an accompanying table-based tool to guide researchers facing difficult decisions about sharing their data.

### 4.2. Implications

Prompted by the current sector-wide move towards open data, this review extracts actionable suggestions from three decades of discourse surrounding qualitative data sharing. The associated resource presents the findings in an accessible table-based format, guiding readers towards the most informative source for each suggestion. This will reduce the time burden associated with locating guidance for future researchers.

The findings highlight the complexity of qualitative data sharing, reiterating the long-standing point that uniform data sharing policies may not adequately address the ethical and practical considerations involved. The findings of the review therefore serve as a call to action for funders and institutions to take these differences into account when developing and applying data sharing requirements or guidelines. In particular, this tool may be used by data repositories as a structure against which to review their policies to support qualitative researchers. For example, Suggestion 1E(iii) of our table-based tool proposes embargoing data, and the shortest time period suggested is 5–10 years. However, ICPSR, at the time of writing, only allows embargo of up to three years. In the meantime, the tool developed from this review may be used to (i) inform qualitative data management plans, (ii) request additional funding for data sharing, or (iii) demonstrate that, after full consideration of the literature, a specific dataset cannot be ethically shared. The review identified a research gap surrounding evaluation. Specifically, few attempts have been made to formally evaluate strategies to safely and usefully share qualitative data. More systematic, transferable evaluation of strategies against predefined success criteria may improve researchers' confidence in the suggestions, thereby encouraging data sharing while reducing the risk of the potential negative effects discussed by the included literature.

Barriers across multiple themes (confidentiality, consent and data misuse) reflect researchers' uncertainty regarding participants' wishes and levels of understanding. This often prevents researchers from making data available, as they assume that participants would want their data to be withheld. However, interviews with participants suggest that for some, data sharing is an expected and valued part of the process. Participants explain that contribution is often motivated by a desire to benefit themselves or their community [4,5,26], and some feel that having multiple researchers analyse their data increases the likelihood of this happening [4]. Clarifying expectations is therefore imperative to protecting participants' interests. The review identifies several suggestions for communicating with participants and verifying their wishes, but the overall pervasiveness of this issue indicates a need for further research on effective two-way communication between qualitative researchers and participants.

### 4.3. Strengths

This review builds on the existing literature surrounding open data by outlining how the principles can be adapted and applied to qualitative data in a non-prescriptive manner. It also highlights a community of dedicated qualitative researchers seeking to engage with open research while maintaining their ethical and philosophical commitments, rather than abandoning or diluting them. The review summarizes the extensive discussions from this community [1,4,7,35,38], presenting them in an accessible, solution-focused format to help facilitate further discussion and practical application. Crucially, the findings of the review and the accompanying tool are applicable to interviews and focus groups across academic disciplines and research contexts.

This work builds on two previous reviews that examined guidance for qualitative data sharing provided by journals [1] and data repositories [11]. Both reviews found limited actionable guidance. By expanding the scope to all outputs, including grey literature, we were able to identify a larger body of relevant material. Crucially, the broader search was also able to capture discussions among qualitative researchers themselves. As a result, this review was able to synthesize more practically relevant suggestions, helping to address the gap identified by previous reviews.

A further strength of the review is that it moves beyond advocating for data sharing as a box-ticking exercise, instead examining how to share qualitative data in ways that genuinely facilitate reuse. Rather than treating sharing as a matter of simply depositing materials in a repository with varied levels of redaction, it highlights how a careful combination of collaboration with participants and contextual documentation can assist future researchers in reuse. This focus is important, as the literature on research data management has increasingly noted that policies promoting data sharing do not necessarily lead to meaningful data reuse, and that substantial social and technical barriers often remain between data creators and potential re-users [48–50]. Importantly, our review encourages researchers to keep future researchers in mind when preparing qualitative data for sharing, so that the materials made available are of the highest possible quality and in the most re-usable state.

### 4.4. Limitations

We acknowledge a number of limitations to our work. First, this review extracted all mentioned strategies from the included sources, regardless of whether these were endorsed or dismissed by the original authors (and without normative evaluation by the present authors). Consequently, some suggestions may appear in the tool despite being rejected as practically possible by the original authors, in their original contexts. In addition, we did not collect the limitations of individual strategies. This is because the strengths and limitations of strategies are highly context-dependent and likely to vary across datasets. The objective of this scoping review is to map existing literature to inform researchers' own evaluation of appropriate strategies for their unique dataset. Collection of evaluative judgements therefore falls outside the scope of the review. However, this means that suggestions may be presented without recognition of their limitations, producing an overly simplistic picture. To mitigate this, the tool links to sources weighing the advantages and disadvantages of suggestions wherever possible. We advise that the review should be used as a starting point, and that readers should read the primary sources reported and use their own critical judgement.

### 4.5. Future research

There remain important questions surrounding the practicalities of qualitative data sharing.

Firstly, the rapid development of artificial intelligence (AI) is likely to influence qualitative data sharing in ways that are not yet fully understood [51–53]. AI may reshape aspects of qualitative analysis and research workflows, and these developments may have implications for how qualitative datasets are prepared, shared and re-used. At present, however, there is limited empirical evidence on how these technologies will ultimately affect data sharing practices. AI could introduce new challenges. For example, increasingly capable systems may make it easier to combine information across multiple datasets or external sources, potentially increasing the risk of reidentification beyond what existing anonymization procedures anticipate. Such capabilities may require researchers and repositories to reconsider current approaches to disclosure risk assessment. Equally, AI may support qualitative data sharing by improving de-identification procedures. AI-assisted tools could help identify context-dependent identifiers that researchers might otherwise overlook, assist with ‘intruder testing’ by systematically searching for identifying information across public sources or support the matching of secondary users to appropriate datasets based on research questions and access conditions. Whether AI ultimately increases or decreases the practical barriers to qualitative data sharing remains an open empirical question. Future research should therefore investigate both the risks and opportunities that AI presents for qualitative data sharing.

Secondly, this review only reports on barriers for which suggestions have been made. Taken in isolation, the results may therefore give the impression that all barriers to qualitative data sharing can be overcome. While the overarching themes we reviewed here are in line with previous literature, there are individual barriers we did not address because no solution is currently described in the literature. For example, Ellis [54] discusses the complexities around protecting not only the participants, but others who are characters in the stories our participants tell us. We found no suggestion in the literature to specifically address this concern. Other examples of concerns that were raised through the literature but did not have any corresponding suggestions were: concerns that the researcher may self-censor the topics they research [55], and that sharing appropriate data may lead to a skewed evidence base in favour of less sensitive narratives [56]. We hope that a researcher concerned about this could use the tool to query archiving mandates by demonstrating that, at present, there have been no suggestions of how to overcome the barrier they are experiencing, and that therefore there exist legitimate reasons not to share data. Future research may examine the extent to which identified barriers are addressed through this review.

Thirdly, it would be beneficial to empirically evaluate the strategies proposed, potentially in context-specific settings. Finally, future research may extend this work beyond interview and focus group transcripts. For example, our initial search revealed a substantive body of literature on clinical notes that we did not address, just as we did not address other types of qualitative documents prevalent in the humanities.

## 5. Conclusion

The movement towards open research has prompted increasing demands for open data, yet guidance for sharing qualitative data remains limited. This scoping review maps actionable suggestions across seven themes of perceived barriers, drawing them together into a structured, accessible resource. In doing so, it highlights both the engaged ongoing discussion and the absence of systematic evaluation of proposed strategies. The literature is clear that guidance must account for the complexity and variety of qualitative data, and must not attempt to impose individual strategies. We intend for this review and the accompanying tool to assist future researchers in more confident, critically informed decision-making surrounding qualitative data management. The review also provides a starting point for researchers seeking to more formally assess qualitative data sharing practices.

## Supplementary Material



## Data Availability

The included sources are available in the supplementary material. The coding applied is accessible as an NVivo output available on the Open Science Framework [57]. Supplementary material is available online [58].
