# Supplementary material for: Barriers to sharing qualitative interview data and suggestions to overcome them: a scoping review
Source: R Soc Open Sci. 2026 Oct 28;13(10):261007. doi: 10.1098/rsos.261007 (PMC13714399; doi:10.1098/rsos.261007)
Supplement: Supplementary file 1 [file rsos.261007.zip › rsos.261007/rsos261007_si_001.docx]

| **Barrier** | **Suggestion** | **Papers** |
| --- | --- | --- |
| Theme 1: Confidentiality | | |
| Uncertainty about what is ‘identifiable information’ | Approach de-identification as an analysis, by dividing transcripts into data points then systematically examining each point to locate identifiers. | *n* = 15  Campbell et al (2023b)  Campbell (2024)  Dodds et al (2020)  DuBois et al (2023)  Endacott et al (2024)  Gupta et al (2021)  John Hopkins Libraries Data Services (2024)  Kapiszeski and Karcher (2021)  Karcher et al (2019)  Kleinberg et al (2017)  Mannheimer et al (2018)  Frohwirth et al (2020)  Saunders et al (2014)  Stewart and Shaffer (2021)  UK Data Service (2024) |
| Insider knowledge | Consult community members during the de-identification process. | *n* = 7  Bishop (2016)  Campbell et al (2019)  Campbell et al (2023a)  Campbell et al (2023a, supplementary material)  Campbell et al (2023b)  Dodds et al (2020)  Gray and Nunn (2023) |
| Uncertainty about how to remediate identifiers | Blurring, by removing the precision, so as to prevent  identification, but retaining important information for future readers. | *n* = 16  Antes et al (2018)  Campbell et al (2023b)  Class et al (2021)  Campbell (2024)  Clark (2006)  Creaven and Kirwan (2025)  Dodds et al (2020, Supplementary material)  DuBois et al (2018)  Dunning and Camp (2015, Anonymisation protocol)  Freidrich et al (2023)  Juros (2022)  McLeod et al (2020)  Meghani et al (2024)  Saunders et al (2014)  Stewart and Shaffer (2021)  UK Data Service (2024) |
|  | Removal | *n* =12  Campbell et al (2023b)  Campbell (2024)  Freidrich et al (2023)  Dunning and Camp (2015, Anonymisation protocol)  Meghani et al (2024) |
|  | Fictionalisation | *n* =12  Bishop (2005)  Clark (2006)  Class et al (2021)  DuBois et al (2018)  Freidrich et al (2023)  Kaiser (2009)  Karhuluhti (2021)  Parry and Mauthner (2006)  Saunders et al (2014)  Slavnic (2011)  Stewart and Shaffer (2021)  UK Data Service (2024) |
| Concern that identifiers may be missed due to human error | Mock intruder testing | *n* = 4  Campbell et al (2023b)  Campbell (2024)  Creaven and Kirwan (2025)  Gray and Nunn (2023) |
|  | Minimise the identifiers collected to begin with | *n* = 2  McGrath and Nilsonne (2018)  UK Data Service (2024) |
|  | Use an established qualitative analysis validity assessment | *n* = 1  Campbell et al (2023b) |
|  | Repository staff may check de-identification | *n* = 1  Corti (2011) |
| Not all data can be de-identified | Impose access controls | *n* = 42  Alexander et al (2020)  Antes et al (2018)  Bishop (2005)  Bishop (2009)  Bishop (2016)  Bishop and Neale (2011)  Branney et al (2022)  Cliggett (2013)  Corti et al (1995)  Corti et al (2000)  Creaven and Kirwan (2025)  Diaz (2021)  Dodds et al (2020)  Dodds et al (2020, Supplementary material)  DuBois et al (2018)  DuBois et al (2023)  Field et al (2021)  Friedrich et al (2023)  Inter-university Consortium for Political and Social Research (2020)  Jones and Alexander (2018)  Juros (2022)  Kapiszeski and Karcher (2021)  Khan et al (2021)  Kirilova and Karcher (2017)  Mannheimer et al (2018)  McCarthy et al (2023)  McGrath and Nilsonne (2018)  McLeod et al (2020)  Meghani et al (2024)  Mozersky et al (2021)  Neale and Bishop (2012a)  Neale and Bishop (2012b)  Frohwirth et al (2020)  Smioski (2011)  Steinhardt et al (2021)  Steinhardt and Kruschick (2022)  Steltenpohl et al (2022)  Stewart and Shaffer (2021)  Tamminen et al (2021)  Turner (2016)  Williams et al (2004)  Yardley et al (2013) |
|  | Embargo data | *n* = 20  Antes et al (2018)  Bishop (2005)  Bishop (2009)  Bishop (2016)  Bishop and Neale (2011)  Corti et al (1995)  Corti et al (2000)  Dodds et al (2020,  Supplementary material)  Inter-university Consortium for Political and Social Research (2020)  Jones and Alexander (2018)  Juros (2022)  Khan et al (2021)  Kirilova and Karcher (2017)  Mannheimer et al (2018)  Neale and Bishop (2012a)  Schubotz et al (2011)  Slavnic (2011)  Slavnic (2013)  Stewart and Shaffer (2021)  Weller (2023) |
|  | Request participant consent to share some identifiers | *n* = 2  Kaiser (2009)  Khan et al (2021)  UK Data Service (2024) |
|  | Share alternative data forms, instead of entire transcripts | *n* = 9  Alexander (2020)  Branney et al (2022)  Campbell et al (2019)  Field et al (2021)  Jones and Alexander (2018)  Khan et al (2021)  Frohwirth et al (2020)  Tamminen et al (2021)  Tsai et al (2016) |
|  | Withold or restrict individual transcripts | *n* = 11  Bishop (2005)  Class et al (2021)  Creaven and Kirwan (2025)  Dodds et al (2020)  Dodds et al (2020, Supplementary material)  DuBois et al (2018)  DuBois et al (2023)  Kaiser (2009)  Stewart and Shaffer (2021)  Weller (2023)  Wutich and Bernard (2016) |
| Theme 2: Consent | | |
| Asking for consent to share data may prevent participation | Opt-in Consent | *n* = 6  Campbell et al (2019)  Campbell et al (2023b)  Gray and Nunn (2023)  Stewart and Shaffer (2021)  Tamminen et al (2021)  VandeVusse et al (2022) |
|  | Explain the benefits of data sharing | *n* = 1  Bishop et al (2017) |
|  | Allow participants to specify access safeguards | *n* = 3  Campbell et al (2019)  Gray and Nunn (2023)  Kapiszeski and Karcher (2019) |
|  | Only ask after data has been collected | *n* = 1  Siegfried (2024) |
|  | Offer member checking | *n* = 2  Gray and Nunn (2023)  McGrath and Nilsonne (2018) |
|  | Offer the option to renew consent at specific points in the future | *n* = 1  Branney et al (2022) |
| Asking for consent to share data may influence what participants disclose | Offer member checking | *n* = 1  Creaven and Kirwan (2025) |
|  | Only ask after data has been collected | *n* = 3  Corti et al (2000)  Neale and Bishop (2012a)  Steinhardt et al (2021) |
| Asking for consent to share data may undermine trust in the researchers | Use restricted access, and make this clear during conversations with participants | *n* = 1  Creaven and Kirwan (2025) |
| Participants may not understand what ‘data sharing’ entails | Develop a verbal script to ensure consistent and considered explanations | *n* = 31  Branney et al (2019)  Branney et al (2022)  Bishop (2009)  Bishop (2016)  Campbell et al (2019)  Campbell et al (2023a, supplementary material)  Campbell et al (2023b)  Carusi and Jirotka (2009)  Class et al (2021)  Corti et al (2000)  DuBois et al (2023)  Inter-university Consortium for Political and Social Research (2020)  Joyce et al (2022)  Karhulahti (2022)  Kaiser (2009)  Khan et al (2021)  McCarthy et al (2023)  McLeod et al (2020)  Méndez de Andés (2023)  Mozersky et al (2021)  Neale and Bishop (2012a)  Frohwirth et al (2020)  Prosser et al (2024)  Saunders et al (2014)  Stegenga et al (2023)  Steltenpohl et al (2025)  Tamminen et al (2021)  UK Data Service (2024)  VandeVusse et al (2022)  Williams et al (2004)  Yardley et al (2013) |
|  | Conduct comprehension checks and do not share transcripts from participants who demonstrate incomplete understanding | *n* = 2  Frohwirth et al (2020)  VandeVusse et al (2022) |
|  | Offer the option to renew consent at specific points in the future | *n* = 2  Kaiser (2009)  Karhulahti (2022)  Kelder (2005)  Yardley et al (2013) |
|  | Offer member checking | *n* = 1  Stewart and Shaffer (2021) |
|  | Embargo data | *n* = 1  McLeod et al (2020) |
|  | Conduct additional common sense risk assessments | *n* = 2  Kirilova and Karcher (2017)  Weller (2023) |
| Participants may agree to data sharing, but share information in the moment that they did not intend to | Discuss with participants what they want to share after data collection has completed | *n* = 9  Campbell et al (2019)  Campbell et al (2023a)  Campbell et al (2023b)  Gore-Gorszewska (2024)  Kaiser (2009)  Karhulahti (2022)  Neale and Bishop (2012a)  Steinhardt et al (2021)  VandeVusse et al (2022) |
| Consent for qualitative data sharing is too complex to be addressed through the traditional, one-off consent form | Treat consent as a process, with steps such as… | *n* = 14  Bishop (2009)  Branney et al (2019)  Campbell et al (2019)  Gore-Gorszewska (2024)  Kaiser (2009)  Kapiszeski and Karcher (2021)  Karhulahti (2022)  Mauthner and Parry (2009)  Neale and Bishop (2012a)  Saunders et al (2014)  Slavnic (2011)  Stewart and Shaffer (2021)  Weller (2023)  Williams et al (2004) |
|  | Notifying participants of the intent to share data prior to data collection | *n* = 2  Campbell et al (2023a)  Mozersky et al (2020) |
|  | Offer the opportunity to discuss data sharing in advance of data collection | *n* = 3  Branney et al (2019)  Karhulahti (2022)  Steinhardt et al (2021) |
|  | Evaluate non-verbal cues from participants | *n* = 1  Karhulahti (2022) |
|  | Discuss data sharing again after data collection | *n* = 3  Branney et al (2019)  Karhulahti (2022)  Steinhardt et al (2021) |
|  | Offer member-checking | *n* = 6  Branney et al (2019)  Field et al (2021)  McGrath and Nilsonne (2018)  Saunders et al (2014)  Steinhardt et al (2021)  Williams et al (2004) |
|  | Offer the opportunity to opt-in to ongoing communication about who is accessing data | *n* = 7  Carusi and Jirotka (2009)  Class et al (2021)  Creaven and Kirwan (2025)  Kaiser (2009)  Karhulahti (2022)  McCarthy et al (2023)  Saunders et al (2014) |
| Theme 3: Context | | |
| Uncertainty about what additional information is required to contextualise transcripts | Use a pre-existing framework of required information | *n* = 8  Antes et al (2018)  Branney et al (2022)  Corti (2000)  Hoyle et al (2012)  Inter-university Consortium for Political and Social Research (2020)  Kuula (2000)  Roller and Lavrakas (2018)  Turner (2016) |
|  | Select from suggested items including: |  |
|  | Associated publications and methodological details | *n* = 26  Antes et al (2018)  Bishop (2006)  Bishop (2016)  Campbell (2024)  DuBois et al (2018)  DuBois et al (2023)  Dunning and Camp (2015)  Fielding (2000)  Haaker (2018)  Inter-university Consortium for Political and Social Research (2020)  Karcher et al (2019)  Karcher et al (2021)  Kjeldgaard (2011)  Kuula (2000)  McLeod et al (2020)  Meghani et al (2024)  Méndez de Andés (2023)  Neale and Bishop (2012a)  Rauf (2022)  Roller and Lavrakas (2018)  Smioski (2011)  Stegenga et al (2023)  Steinhardt et al (2021)  Steltenpohl et al (2022)  van den Berg (2008)  Walters (2009) |
|  | Consent forms | *n* = 4  Bishop (2006)  Inter-university Consortium for Political and Social Research (2020)  McLeod et al (2020)  Stewart and Shaffer (2021) |
|  | Contextual summary | *n* = 8  Corti (2018)  Corti (2011)  Dunning and Camp (2015)  Karcher et al (2019)  McLeod et al (2020)  Neale and Bishop (2012a)  Steinhardt and Kruschick (2022)  Steltenpohl et al (2022) |
|  | Correspondence with sponsors | *n* = 1  Fielding (2000) |
|  | Dialect glossary | *n* = 1  Corti (2011) |
|  | Ethics applications | *n* = 1  McLeod et al (2020) |
|  | Details of the de-identification process | *n* = 10  Antes et al (2018)  Bishop (2006)  Campbell (2024)  DuBois et al (2023)  Inter-university Consortium for Political and Social Research (2020)  Karcher et al (2019)  Kirilova and Karcher (2017)  McLeod et al (2020)  Neale and Bishop (2012a)  Steinhardt et al (2021) |
|  | Information about individual participants | *n* = 7  Bishop (2006)  DuBois et al (2023)  Inter-university Consortium for Political and Social Research (2020)  Khan et al (2021)  Neale and Bishop (2012a)  Steinhardt et al (2021)  Stewart and Shaffer (2021) |
|  | Information about the participant/interviewer relationship | *n* = 6  Bishop (2006)  van den Berg (2008) |
|  | Reflexivity notes | *n* = 32  Antes et al (2018)  Bishop (2006)  Branney et al (2019)  Campbell (2024)  Cheshire et al (2009)  Corti (2000)  Corti (2011)  Corti and Thompson (1998)  DuBois et al (2018)  Field et al (2021)  Fielding (2000)  Feldman and Shaw (2019)  Gore-Gorszewska (2024)  Haaker (2018)  Inter-university Consortium for Political and Social Research (2020)  Jones and Alexander (2018)  Kapiszeski and Karcher (2021)  Karcher et al (2019)  Karcher et al (2021)  Kelder (2005)  McLeod et al (2020)  Neale and Bishop (2012a)  Neale and Bishop (2012b)  Nettleton (2018)  Rauf (2022)  Roller and Lavrakas (2018)  Smioski (2011)  Stegenga et al (2023)  Steinhardt et al (2021)  Steltenpohl et al (2022)  Tamminen and Poucher (2018)  Walters (2009) |
|  | Interview setting | *n* = 13  Bishop (2006)  DuBois et al (2023)  Field et al (2021)  Inter-university Consortium for Political and Social Research (2020)  Karcher et al (2019)  Kuula (2000)  Meghani et al (2024)  Neale and Bishop (2012b)  Roller and Lavrakas (2018)  Stegenga et al (2023b)  Steinhardt et al (2021)  van den Berg (2008)  Walters (2009) |
|  | Timelines | *n* = 4  Bishop (2006)  Meghani et al (2024)  Neale and Bishop (2012a)  Stegenga et al (2023) |
|  | Topic Guides | *n* = 14  Antes et al (2018)  Bishop (2006)  Bishop (2016)  DuBois et al (2018)  DuBois et al (2023)  Field et al (2021)  Fielding (2000)  Inter-university Consortium for Political and Social Research (2020)  Karcher et al (2019)  Karcher et al (2021)  McLeod et al (2020)  Roller and Lavrakas (2018)  Steinhardt et al (2021)  Stewart and Shaffer (2021) |
| De-identifying data may change the underlying meaning | Explicitly note the impact of de-identification | *n* = 3  Antes et al (2018)  Elman et al (2010)  UK Data Service (2024) |
|  | Ask for permission to share some identifiers | *n* = 2  Antes et al (2018)  Walsh et al (2025) |
|  | Select only the most contextually relevant identifiers to retain | *n* = 5  Dodds et al (2020)  Dodds et al (2020, Supplementary material)  DuBois et al (2018)  Friedrich et al (2023)  Saunders et al (2014) |
|  | Impose access controls | *n* = 10  Class et al (2021)  Corti et al (2000)  Dodds et al (2020, Supplementary material)  DuBois et al (2023)  Fielding (2000)  Friedrich et al (2023)  McCarthy et al (2023)  Neale and Bishop (2012a)  Stegenga et al (2023)  Steltenpohl et al (2022) |
| Context cannot be portrayed through notes | Start each interview with contextual questions | *n* = 1  Rauf (2022) |
|  | Promote collaboration with the primary researchers | *n* = 10  Diaz (2021)  DuBois et al (2023)  Feldman and Shaw (2019)  Friedrich et al (2023)  Field et al (2021)  Gore-Gorszewska (2024)  Meghani et al (2024)  Mozersky et al (2020)  Weller (2023)  Yardley et al (2013) |
| Theme 4: Data Misuse | | |
| Consult experts or insiders to develop the data management plan | | *n* = 1  Hardy et al (2016) |
| Encourage contact with the primary researchers | | *n* = 3  Branney et al (2019)  Gore-Gorszewska (2024)  Mozersky et al (2020) |
| Impose access controls | | *n* = 15  Alexander et al (2020)  Bishop (2005)  Corti et al (2000)  DuBois et al (2018)  DuBois et al (2023)  Gore-Gorszewska (2024)  Gray and Nunn (2023)  Hardy et al (2016)  Jones and Alexander (2018)  Kjeldgaard (2011)  Mozersky et al (2020)  Neale and Bishop (2012b)  Tamminen and Poucher (2018)  VandeVusse et al (2022)  Yardley et al (2013) |
| Require participant consent for each access request | | *n* = 3  Branney et al (2022)  Neale and Bishop (2012b)  Williams et al (2004) |
| Theme 5: External Permissions | | |
| Copyright | Ask participants to sign over copyright | *n* = 7  Branney et al (2019)  Kapiszeski and Karcher (2021)  Mannheimer et al (2018)  Neale and Bishop (2012a)  Parry and Mauthner (2004)  Smioski (2011)  Williams et al (2004) |
|  | Fair use exception | *n* = 2  Mannheimer et al (2018)  Parry and Mauthner (2004) |
| Ethical approval | The four step approach to discussing qualitative data sharing with ethical panels | *n* = 1  Kaiser (2009) |
| Theme 6: Researcher Distress | | |
| Feelings of moral responsibility | Discuss with participants | *n* = 1  Williams et al (2004) |
|  | Conduct additional common sense risk assessment | *n* = 2  Stewart and Shaffer (2021)  Weller (2023) |
| Vicarious trauma | Enforced shifts for team members | *n* = 1  Campbell (2024) |
|  | Signposting to support | *n* = 2  Campbell et al (2023b)  Campbell (2024) |
|  | Team-member check-ins | *n* = 1  Campbell et al (2023b) |
| There’s nowhere to get help | Academic librarians | *n* = 4  Creaven and Kirwan (2025)  Friedrich et al (2023)  Mannheimer et al (2018)  Meghani et al (2024) |
|  | Data management experts | *n* = 3  Creaven and Kirwan (2025)  Kirilova and Karcher (2017)  Meghani et al (2024) |
|  | Institutional review boards | *n* = 1  Walsh et al (2025) |
|  | IT departments | *n* = 1  Meghani et al (2024) |
|  | Participants | *n* = 1  Campbell et al (2019) |
|  | Repository staff | *n* = 15  Bishop (2005)  Bishop (2016)  Campbell (2024)  Cheshire et al (2009)  Corti et al (1995)  Corti and Blackhouse (2005)  Dodds et al (2020)  Inter-university Consortium for Political and Social Research (2020)  Karcher et al (2019)  Kirilova and Karcher (2017)  Mannheimer et al (2018)  Meghani et al (2024)  Mozersky et al (2021)  Steinhardt et al (2021)  Stewart and Shaffer (2021) |
|  | Steering groups | *n* = 1  Dodds et al (2020) |
|  | Subject matter experts | *n* = 2  Campbell (2024)  Stewart and Shaffer (2021) |
| Theme 7: Time and Money | | |
|  | Archives may take some de-identification work | *n* = 3  Corti (2011)  Meghani et al (2024)  Smioski (2011) |
|  | Ask for help | *n* = 1  Mannheimer et al (2018) |
|  | Ask for permission to share some identifiers | *n* = 2  Corti and Blackhouse (2005)  Frohwirth et al (2020) |
|  | Automated de-identification | *n* = 19  Campbell et al (2023b)  Campbell (2024)  Class et al (2021)  Cliggett (2013)  DuBois et al (2018)  DuBois et al (2023)  The Stanford Natural Language Processing Group (2024)  Gupta et al (2021)  John Hopkins Libraries Data Services (2024)  Kaiser (2009)  Kirilova and Karcher (2017)  Kleinberg et al (2017)  McLeod et al (2020)  Mozersky et al (2021)  Ó Dúlaigh (2013)  Stewart and Shaffer (2021)  UK Data Service (2022)  UK Data Service (2024)  Walsh et al (2025) |
|  | Factor the time and financial requirements into grant applications | *n* = 20  Alexander et al (2020)  Campbell et al (2023b)  Corti et al (1995)  Corti et al (2000)  Diaz (2021)  Dodds et al (2020)  DuBois et al (2018)  DuBois et al (2023)  Hardy et al (2016)  Mannheimer et al (2018)  McCarthy et al (2023)  Meghani et al (2024)  Neale and Bishop (2012a)  Neale and Bishop (2012b)  Nettleton (2018)  Saunders et al (2014)  Slavnic (2011)  Slavnic (2013)  Stewart and Shaffer (2021)  Weller (2023) |
|  | Submit handwritten field notes | *n* = 1  Corti et al (1995) |
|  | Increase access controls | *n* = 2  Mannheimer et al (2018)  Stewart and Shaffer (2021) |
|  | Plan de-identification process in advance | *n* = 7  Antes et al (2018)  Campbell (2024)  Dodds et al (2020)  DuBois et al (2018)  Inter-university Consortium for Political and Social Research (2020)  Kapiszeski and Karcher (2021)  Stewart and Shaffer (2021) |
|  | Share a selection of transcripts | *n* = 1  Kirilova and Karcher (2017) |
|  | Share coding queries | *n* = 1  Gore-Gorszewska (2024) |

Full list of included studies:

Alexander SM, Jones K, Bennett NJ, Budden A, Cox M, Crosas M, Game ET, Geary J, Hardy RD, Johnson JT and Karcher S, 2020. Qualitative data sharing and synthesis for sustainability science. *Nature Sustainability*, **3**, 81–88

Antes AL, Walsh HA, Strait M, Hudson-Vitale CR, DuBois JM. Examining data repository guidelines for qualitative data sharing. 2018. *Journal of Empirical Research on Human Research Ethics*. **13**, 61-73. (doi: [10.1177/1556264617744121](https://doi.org/10.1177/1556264617744121))

Bishop L, 2005. Protecting respondents and enabling data sharing: Reply to Parry and Mauthner. *Sociology*, **39**, 333–336 (doi: 10.1177/0038038505050542)

Bishop L, 2006. A proposal for archiving context for secondary analysis. *Methodological Innovations Online*, **1**, 10–20. (doi: [10.4256/mio.2006.0008](https://doi.org/10.4256/mio.2006.0008))

Bishop L, 2009. Ethical sharing and reuse of qualitative data. *Australian Journal of Social Issues*, **44**, 255–272 (doi: 10.1002/j.1839-4655.2009.tb00145.x)

Bishop L, 2016. Sharing Qualitative Data - Challenges and Opportunities (Accessed via: <https://www.slideshare.net/slideshow/sharing-qualitative-data-challenges-and-opportunities/74878104>)

Bishop L, 2017. Supplementary material for Sharing Qualitative Data - Challenges and Opportunities (Accessed via: <https://www.slideshare.net/slideshow/sharing-qualitative-data-challenges-and-opportunities/74878104>)

Bishop L and Neale B, 2011. Sharing qualitative and qualitative longitudinal data in the UK. *IASSIST Quarterly*, **34**, 23

Branney PE, Brooks J, Kilby L, Newman K, Norris E, Pownall M, Talbot CV, Treharne GJ and Whitaker CM, 2023. Three steps to open science for qualitative research in psychology. *Social and Personality Psychology Compass*, **17**. ([https://doi.org/10.1111/spc3.12728](https://psycnet.apa.org/doi/10.1111/spc3.12728))

Branney P, Reid K, Frost N, Coan S, Mathieson A, Woolhouse M. 2019. A context-consent meta-framework for designing open (qualitative) data studies. *Qualitative Research in Psychology.* **16,** 483–502**.** (doi: [10.1080/14780887.2019.1605477](https://doi.org/10.1080/14780887.2019.1605477?urlappend=%3Futm_source%3Dresearchgate.net%26utm_medium%3Darticle))

Campbell R, 2024. Sharing Sensitive Qualitative Data. (Accessed via https://www.youtube.com/watch?v=-Pha3EINF3s)

Campbell R, Goodman-Williams R and Javorka M, 2019. A trauma-informed approach to sexual violence research ethics and open science. *Journal of Interpersonal Violence*, **34**, 4765–4793 (doi: 10.1177/0886260519871530)

Campbell R, Goodman-Williams R, Engleton J, Javorka M, Gregory K. 2023a. Open science and data sharing in trauma research: Developing a trauma-informed protocol for archiving sensitive qualitative data. *Psychological Trauma: Theory, Research, Practice, and Policy*. 34, (doi: [https://doi.org/10.1037/tra0001358](https://psycnet.apa.org/doi/10.1037/tra0001358))

Campbell R, Goodman-Williams R, Engleton J, Javorka M, Gregory K. 2023. Supplementary material for Open science and data sharing in trauma research: Developing a trauma-informed protocol for archiving sensitive qualitative data. (Accessed via https://suapa.org/psycarticles/supplemental/tra0001358/tra0001358_suhtml)

Campbell R, Javorka M, Engleton J, Fishwick K, Gregory K and Goodman-Williams R, 2023b. Open-science guidance for qualitative research: An empirically validated approach for de-identifying sensitive narrative data. *Advances in Methods and Practices in Psychological Science*, **6**, 25152459231205832 (doi: <https://doi.org/10.1177/25152459231205832>)

Carusi A and Jirotka M, 2009. From data archive to ethical labyrinth. *Qualitative Research*, **9**, 285–298 (doi: 10.1177/1468794109105032)

Cheshire L, Broom A and Emmison M, 2009. Archiving qualitative data in Australia: An introduction. *The Australian Journal of Social Issues*, **44**, 239–254. (doi: [10.1002/j.1839-4655.2009.tb00144.x](https://doi.org/10.1002/j.1839-4655.2009.tb00144.x))

Clark. 2006. Anonymising Research Data. (Accessed via <https://www.researchgate.net/publication/277877831_Anonymising_Research_Data>)

Class B, de Bruyne M, Wuillemin C, Donzé D and Claivaz JB, 2021. Towards Open Science for the Qualitative Researcher: From a Positivist to an Open Interpretation. *International Journal of Qualitative Methods*, **20**, 1–15. (doi: <https://doi.org/10.1177/16094069211034641>)

Cliggett L, 2013. Qualitative data archiving in the digital age: Strategies for data preservation and sharing. *The Qualitative Report*, **18**

Corti L, 2011. The European landscape of qualitative social research archives: Methodological and practical issues. In *Forum: Qualitative Social Research*, **12**

Corti L, 2018. Show me the data: research reproducibility for qualitative methods. (Accessed via: https://blog.ukdataservice.ac.uk/show-me-the-data/)

Corti L and Backhouse G, 2005, May. Acquiring qualitative data for secondary analysis. In *Forum Qualitative Sozialforschung*, **6**.

Corti L, Day A and Backhouse G, 2000, December. Confidentiality and informed consent: Issues for consideration in the preservation of and provision of access to qualitative data archives. In *Forum Qualitative Sozialforschung/Forum: Qualitative Social Research*, **1**

Corti L, Foster J and Thompson P. 1995. *Archiving qualitative research data*. (Accessed on 5^th^ June, 2024 via [https://sru.soc.surrey.ac.uk/SRU10.html)](https://dataservices.library.jhu.edu/resources/applications-to-assist-in-de-identification-of-human-subjects-research-data/)

Corti L and Thompson P, 1998. Are you sitting on your qualitative data? Qualidata's mission. *International Journal of Social Research Methodology*, **1**, 85–89 (doi: 10.1080/13645579.1998.10846865)

Creaven AM, Kirwan EM. 2025. Open qualitative data: a worked example of qualitative data sharing from an interview study on loneliness in young adulthood. *Qualitative Research in Psychology*. **22**, 1-25. (doi: [10.1080/14780887.2024.2423923](https://doi.org/10.1080/14780887.2024.2423923?urlappend=%3Futm_source%3Dresearchgate.net%26utm_medium%3Darticle))

Diaz P, 2021. *Archiving Qualitative Data in Practice: Ethical Feedback*. *Bulletin de Méthodologie Sociologique*, **150**, 7–27 (doi: 10.1177/0759106321995678)

Dodds C, Keogh P, Bourne A, McDaid L, Squire C, Weatherburn P, Young I. 2021. The long and winding road: Archiving and re-using qualitative data from 12 research projects spanning 16 years. *Sociological Research Online.* **26**, 269-287. (fdoi:  
https://doi.org/10.1177/1360780420924044)

Dodds C, Keogh P, Bourne A, McDaid L, Squire C, Weatherburn P and Young I, 2021. Supplemental material for: The Long and Winding Road: Archiving and Re-Using Qualitative Data from 12 Research Projects Spanning 16 Years. *SAGE Journals* (Dataset, V1) (doi: 10.25384/SAGE.12489428.v1)

DuBois JM, Mozersky J, Parsons M, Walsh HA, Friedrich A and Pienta A, 2023. Exchanging words: Engaging the challenges of sharing qualitative research data. *Proceedings of the National Academy of Sciences*, **120**, e2206981120 (doi: 10.1073/pnas.2206981120)

DuBois JM, Strait M, Walsh H. 2018. Is it time to share qualitative research data?. *Qualitative psychology*. **5**, 380-393. (doi: 10.1037/qup0000076)

Dunning T and Camp E, 2015. Brokers, voters, and clientelism: The puzzle of distributive politics. *Qualitative Data Repository* (QDR Main Collection, V1) (doi: 10.5064/F6Z60KZB)

Dunning T and Camp E, 2015. Anonymisation protocol for: Brokers, voters, and clientelism: The puzzle of distributive politics. (Accessed via: <https://data.qdr.syr.edu/dataset.xhtml?persistentId=doi:10.5064%2FF6Z60KZB&utm>)

Elman C, Kapiszewski D and Vinuela L, 2010. Qualitative data archiving: Rewards and challenges. *PS: Political Science and Politics*, **43**, 23–27 (doi: 10.1017/S104909651099077X)

Endacott C, Reed B and Steltenpohl CN. Embracing open science in qualitative organizational research: Extant areas of integration and future possibilities (doi: 10.31234/osf.io/vzkdp)

Feldman S and Shaw L, 2019. The epistemological and ethical challenges of archiving and sharing qualitative data. *American Behavioral Scientist*, **63**, 699–721 (doi: 10.1177/0002764218796084)

Field SM, van Ravenzwaaij D, Pittelkow MM, Hoek JM and Derksen M, 2021. Qualitative open science–pain points and perspectives (doi: 10.31219/osf.io/e3cq4)

Fielding N, 2000. The shared fate of two innovations in qualitative methodology: The relationship of qualitative software and secondary analysis of archived qualitative data. *Forum: Qualitative Social Research*, **1**.

Friedrich AB, Mozersky J and DuBois JM, 2023. Potentially identifying variables reported in 100 qualitative health research articles: Implications for data sharing and secondary analysis. In *Forum Qualitative Sozialforschung/Forum: Qualitative Social Research*, **24** (doi: 10.1177/16094069221105074)

Frohwirth L, Karcher S and Mueller J, 2020. Theory and Practice of Transparent Qualitative Health Research. (Accessed 7^th^ November 2024 via: https://www.youtube.com/watch?v=DKkExQfwqpU)

Gore-Gorszewska G, 2024. “I’m telling you my story, not publishing a blog”: Considerations and suggestions on data sharing in qualitative health psychology research on sensitive topics. *Journal of Health Psychology*, **29**, 665–673 (doi: 10.1177/13591053241239109)

Gray R and Nunn J, 2023. Data sharing in qualitative research. *Nurse Author & Editor*, **33**, 4–5 (doi: 10.1111/nae2.12049)

Gupta A, Lai A, Mozersky J, Ma X, Walsh H and DuBois JM, 2021. Enabling qualitative research data sharing using a natural language processing pipeline for deidentification: moving beyond HIPAA Safe Harbor identifiers. *JAMIA Open*, **4**, ooab069 (doi: 10.1093/jamiaopen/ooab069)

Haaker M, 2018. Some practical protocols for transparency for qualitative research (Accessed via: https://ukdataservice.ac.uk/app/uploads/rdm_transparency_2020-01-24.pdf)

Hardy LJ, Hughes A, Hulen E, Schwartz AL. 2016. Implementing qualitative data management plans to ensure ethical standards in multi-partner centers. *Journal of Empirical Research on Human Research Ethics*. **11**, 191-198. (doi: [10.1177/1556264616636233](https://doi.org/10.1177/1556264616636233))

Hoyle L and Corti L, Gregory A, Martinez A, Wackerow J, Alvar E, Betancort Cabrera N, Gallagher D, Gebel T, Hautamaki J, Kuula A, McEachern S and Zuell C. A Qualitative Data Model for DDI. (Accessed on 5^th^ June, 2024 via <https://ddialliance.org/working-paper-series>)

Inter-university Consortium for Political and Social Research, 2005. Guide to social science data preparation and archiving. (Accessed via: https://www.icpsr.umich.edu/sites/icpsr/manage-data/prepare-deposit/guide)

John Hopkins Libraries. Applications to Assist in De-identification of Human Subjects Research Data. (Accessed on 5^th^ June, 2024 via [https://dataservices.library.jhu.edu/resources/applications-to-assist-in-de-identification-of-human-subjects-research-data/)](https://dataservices.library.jhu.edu/resources/applications-to-assist-in-de-identification-of-human-subjects-research-data/)

Jones K, Alexander SM, Bennett N, Bishop L, Budden A, Cox M, Crosas M, Game E, Geary J, Hahn C and Hardy D, 2018. Qualitative data sharing and re-use for socio-environmental systems research: A synthesis of opportunities, challenges, resources and approaches (doi: 10.13016/M2WH2DG59)

Joyce JB, Douglass T, Benwell B, Rhys CS, Parry R, Simmons R and Kerrison A, 2023. Should we share qualitative data? Epistemological and practical insights from conversation analysis. *International Journal of Social Research Methodology*, **26**, 645–659. (doi: <https://doi.org/10.1080/13645579.2022.2087851>)

Juros TV, 2022. Challenges of qualitative data sharing in social sciences. *European Science Editing*, **48**, e77781 (doi: 10.3897/ese.2022.e77781)

Kaiser K. 2009. Protecting respondent confidentiality in qualitative research. *Qualitative health research.* **19**, 1632-1641. (doi: [10.1177/1049732309350879](https://doi.org/10.1177/1049732309350879))

Kapiszewski D and Karcher S, 2019. Openness in practice in qualitative research (doi: 10.33774/apsa-2019-if2he)

Kapiszewski D and Karcher S, 2021. Transparency in practice in qualitative research. *PS: Political Science & Politics*, **54**, 285–291. (doi: <https://doi.org/10.1017/S1049096520000955>)

Karcher S, Kirilova D and Page C, 2019. Sharing and managing qualitative data. (Accessed via: https://surface.syr.edu/miga/2)

Karcher S, Kirilova D, Pagé C and Weber N, 2021. How data curation enables epistemically responsible reuse of qualitative data. *The Qualitative Report* (doi: 10.46743/2160-3715/2021.5012)

Karhulahti VM, 2023. Reasons for qualitative psychologists to share human data. *British Journal of Social Psychology*, **62**, 1621–1634 (doi: 10.1111/bjso.12573)

Kelder JA, 2005. Using someone else's data: problems, pragmatics and provisions

Khan J, Rantanen VET and Kolmonen PT, 2021. Open access to qualitative research data: A guide for PhD students in the social sciences at the University of Helsinki. (Accessed via: https://researchportal.helsinki.fi/en/publications/open-access-to-qualitative-research-data-a-guide-for-phd-students/)

Kirilova D and Karcher S, 2017. Rethinking data sharing and human participant protection in social science research: Applications from the qualitative realm. *Data Science Journal*, **16**, 43

Kjeldgaard ASF, 2011. Archiving and disseminating qualitative data in Denmark. *IASSIST Quarterly*, **34**, 38–38. (doi: [10.29173/iq460](https://doi.org/10.29173/iq460))

Kleinberg B, Mozes M, van der Toolen Y and Verschuere B, 2017. Netanos–named entity-based text anonymization for open science. *Preprint at OSF* (doi: 10.31219/osf.io/w9nhb)

Kuula A, 2000. Making qualitative data fit the "Data Documentation Initiative" or vice versa?. In *Forum Qualitative Sozialforschung/Forum: Qualitative Social Research*, **1**

Kuula A, 2011. Methodological and ethical dilemmas of archiving qualitative data. *IASSIST Quarterly*, **34**, 12–12

Mannheimer S, Pienta A, Kirilova D, Elman C, and Wutich A. 2019. Qualitative data sharing: Data repositories and academic libraries as key partners in addressing challenges. *American Behavioral Scientist*. **63,** 643-664.

Mauthner NS and Parry O, 2009. Qualitative data preservation and sharing in the social sciences: On whose philosophical terms? *Australian Journal of Social Issues*, **44**, 291–307 (doi: 10.1002/j.1839-4655.2009.tb00147.x)

McCarthy M, Gillies K, Rousseau N, Wade J, Gamble C, Toomey E, Matvienko-Sikar K, Sydes M, Dowling M, Bryant V, Biesty L. 2023. Qualitative data sharing practices in clinical trials in the UK and Ireland: towards the production of good practice guidance. *HRB open research* **6**. (doi: [10.12688/hrbopenres.13667.1](https://doi.org/10.12688/hrbopenres.13667.1))

McGrath C and Nilsonne G, 2018. Data sharing in qualitative research: Opportunities and concerns. *MedEdPublish*, **7**, 255 (doi: 10.15694/mep.2018.0000255.1)

McLeod J, O’Connor K and Davis N, 2020. Doing research differently: archiving and sharing qualitative data in studies of childhood, education and youth. (doi: 10.25916/5e9e28eec21a1)

Meghani S, Mooney-Doyle K, Barnato, A, Colborn K, Gillette R, Harrison K, Hinds P, Kirilova D, Knafl, K, Schulman-Green D, Pollak K. 2024. Lessons learned establishing the Palliative Care Research Cooperative's qualitative data repository. *Journal of pain and symptom management* **68**, 308-318.  ([10.1016/j.jpainsymman.2024.05.027](https://doi.org/10.1016/j.jpainsymman.2024.05.027))

Mendez De Andes Aldama A, 2023. Open Research Case Study: Ana Méndez de Andés-Using the Open Science Framework to share research data and contextual materials for a study on urban commons. (<https://doi.org/10.15131/shef.data.23702310>)

Mozersky J, McIntosh T, Walsh HA, Parsons MV, Goodman M and DuBois JM, 2021. Barriers and facilitators to qualitative data sharing in the United States: A survey of qualitative researchers. *PLoS One*, **16**, e0261719 (doi: 10.1371/journal.pone.0261719)

Neale B and Bishop L, 2012a. The Timescapes archive: A stakeholder approach to archiving qualitative longitudinal data. *Qualitative Research*, **12**, 53–65.

Neale B and Bishop L, 2012b. The ethics of archiving and re-using qualitative longitudinal data: A stakeholder approach. Timescapes Methods Guide Series, 18.

Nettleton S, 2018. CRISIS WHAT CRISIS: Can qualitative data archiving enhance transparency? (Accessed via: https://ukdataservice.ac.uk/app/uploads/nettleton__rmf_qualtransperency_bath2018.pdf)

Ó Dúlaigh K, 2013. IQDA Qualitative Data Anonymizer. (Accessed via: <https://sourceforge.net/projects/datatool/>)

Parry O, Mauthner NS. 2004. Whose data are they anyway? Practical, legal and ethical issues in archiving qualitative research data. *sociology*. **38**, 139-152. (doi:  
https://doi.org/10.1177/0038038504039366)

Prosser AB, Bagnall R, Higson-Sweeney N. 2024. Reflection over compliance: Critiquing mandatory data sharing policies for qualitative research. *Journal of Health Psychology* **29**, 653-658. (doi:[10.1177/13591053231225903](https://doi.org/10.1177/13591053231225903))

Rauf A, 2022. Making the case for reusing and sharing data in qualitative consumer research. *Consumption Markets & Culture*, **25**, 159–175 (doi: 10.1080/10253866.2021.1987226)

Roller MR and Lavrakas PJ, 2018. A total quality framework approach to sharing qualitative research data: Comment on Dubois et al. (2018). *Qualitative Psychology,* **5**, 394-401. (<https://doi.org/10.1037/qup0000081>)

Saunders B, Kitzinger J, Kitzinger C. 2015. Anonymising interview data: Challenges and compromise in practice. *Qualitative research.* **15**, 616-32. (doi: [10.1177/1468794114550439](https://doi.org/10.1177/1468794114550439))

Schubotz D, Melaugh M and McLoughlin P, 2011, September. Archiving qualitative data in the context of a society coming out of conflict: Some lessons from Northern Ireland. In *Forum: Qualitative Social Research*, **12**.

Siegfried D, 2024. Qualitative business data opened up for subsequent use: A pilot test. (Accessed on 9^th^ April, 2025 via https://open-science-future.zbw.eu/en/qualitative-business-data-subsequent-use-pilot-test/)

Slavnic Z, 2011. Preservation and sharing of qualitative data: Academic debate and policy developments. *Linköping University Electronic Press* (Accessed via: <https://www.diva-portal.org/smash/record.jsf?pid=diva2%3A462823&dswid=2142>)

Slavnic Z, 2013, May. Towards qualitative data preservation and re-use-policy trends and academic controversies in UK and Sweden. In *Forum: Qualitative Social Research*, **14**.

Smioski A, 2011. Archiving qualitative data: Infrastructure, acquisition, documentation, distribution—experiences from WISDOM, the Austrian Data Archive. In *Forum Qualitative Sozialforschung/Forum: Qualitative Social Research*, **12**

Stegenga SM, Steltenpohl CN, Lustick H, Meyer MS, Renbarger R, Standiford Reyes L and Lee LE, 2024. Qualitative research at the crossroads of open science and big data: Ethical considerations. *Social and Personality Psychology Compass*, **18**, p.e12912. (doi: https://doi.org/10.1111/spc3.12912)

Steinhardt I, Fischer C, Heimstädt M, Hirsbrunner SD, Ikiz-Akinci D, Kressin L, Kretzer S, Möllenkamp A, Porzelt M, Rahal RM and Schimmler S, 2021. Opening up and sharing data from qualitative research: A primer (doi: 10.17620/02671.63)

Steinhardt I and Kruschick F, 2022. Knowledge Equity and Open Science in qualitative research–Practical research considerations. *Research Ideas and Outcomes*, **8**, e86387 (doi: 10.3897/rio.8.e86387)

Steltenpohl CN, Lustick H, Meyer MS, Lee LE, Stegenga SM, Reyes LS and Renbarger RL, 2023. Rethinking transparency and rigor from a qualitative open science perspective. *Journal of Trial and Error*, **5**, 47–59

Steltenpohl CN, Mawhinney B, Karhulahti VM and Prosser AM, 2025. Open science for qualitative research. *BMJ Evidence-Based Medicine*, **30**, 413–419 (doi: 10.1136/bmjebm-2024-113532)

Stewart E and Shaffer M, 2021. The Ethical and Practical Challenges of Archiving Refugee Accounts: Reflections from Two Research Projects in the UK. *Bulletin de Méthodologie Sociologique*, **150**, 51–69. (doi: https://doi.org/10.1177/0759106321995707)

Tamminen KA, Bundon A, Smith B, McDonough MH, Poucher ZA and Atkinson M, 2021. Considerations for making informed choices about engaging in open qualitative research. *Qualitative Research in Sport, Exercise and Health*, **13**, 864–886. (doi: <https://doi.org/10.1080/2159676X.2021.1901138>)

Tamminen KA and Poucher ZA, 2018. Open science in sport and exercise psychology: Review of current approaches and considerations for qualitative inquiry. *Psychology of Sport and Exercise*, **36**, 17–28 (doi: 10.1016/j.psychsport.2017.12.010)

The Stanford Natural Language Processing Group, 2024. (Accessed on 13^th^ August 2024 via: https://nlp.stanford.edu/software/CRF-NER.shtml)

Tsai AC, Kohrt BA, Matthews LT, Betancourt TS, Lee JK, Papachristos AV, Weiser SD, Dworkin SL. 2016. Promises and pitfalls of data sharing in qualitative research. Social Science & Medicine. **1**, 191-198. (doi: [10.1016/j.socscimed.2016.08.004](https://doi.org/10.1016/j.socscimed.2016.08.004))

Turner D, 2019. Archiving qualitative data: will secondary analysis become the norm? (Accessed on 5^th^ June, 2024 via [https://ukdataservice.ac.uk/learning-hub/research-data-management/anonymisation/anonymising-qualitative-data/)](https://www.quirkos.com/blog/post/qualitative-archives-secondary-analysis-software/)

UK Data Service.2022. Text Anonymisation Helper Tool: User Guide. (Accessed via <https://ukdataservice.ac.uk/learning-hub/research-data-management/anonymisation/anonymising-qualitative-data/>)

UK Data Service. 2024. Anonymising Qualitative Data. (Accessed on 5^th^ June, 2024 via <https://ukdataservice.ac.uk/learning-hub/research-data-management/anonymisation/anonymising-qualitative-data/>)

Van den Berg H, 2008. Reanalyzing qualitative interviews from different angles: The risk of decontextualization and other problems of sharing qualitative data. *Historical Social Research/Historische Sozialforschung*, 179–192

VandeVusse A, Mueller J and Karcher S, 2022. Qualitative data sharing: Participant understanding, motivation, and consent. *Qualitative Health Research*, **32**, 182–191 (doi: 10.1177/1049732321105405)

Walsh HA, Parsons MV, Mozersky J, Gupta A, Lai AM, Friedrich AB and DuBois JM, 2025. Responsible Sharing of Qualitative Research Data: Insights From a Pioneering Project in the United States. *International Journal of Qualitative Methods*, **24**, 1–13. (doi: https://doi.org/10.1177/16094069251329607)

Walters P, 2009. Qualitative archiving: Engaging with epistemological misgivings. *Australian Journal of Social Issues*, **44**, 309–320 (doi: 10.1002/j.1839-4655.2009.tb00148.x)

Weller S. 2023. Fostering habits of care: Reframing qualitative data sharing policies and practices. *Qualitative Research.* **23**, 1022-1041. (doi: [10.1177/14687941211061054](http://doi.org/10.1177/14687941211061054))

Williams ML, Dicks B, Coffey AJ and Mason B, 2004. Qualitative data sharing and reuse: Mapping the ethical terrain. Briefing paper (Accessed via: <https://orca.cardiff.ac.uk/id/eprint/60704/>)

Wutich A and Bernard HR, 2016. Sharing qualitative data & analysis. With whom and how widely?: A response to ‘Promises and pitfalls of data sharing in qualitative research’. *Social Science & Medicine*, **169**, 199–200. (doi: <https://doi.org/10.1016/j.socscimed.2016.09.041>)

Yardley SJ, Watts KM, Pearson J, Richardson JC. 2014. Ethical issues in the reuse of qualitative data: Perspectives from literature, practice, and participants. *Qualitative Health Research.* **24**, 102-13. (doi: [10.1177/1049732313518373](https://doi.org/10.1177/1049732313518373))
